# Air embolism caused by peripheral superficial vein catheterization: A case report

**DOI:** 10.1097/MD.0000000000037640

**Published:** 2024-04-05

**Authors:** Xiaoxiao Zhou, XingMing Zhong, Liying Dong

**Affiliations:** a The Department of Neurosurgery, The First People’s Hospital of Huzhou, Zhejiang, China; b The Department of Nursing, The First People’s Hospital of Huzhou, Zhejiang, China.

**Keywords:** air embolism, case report, high flow oxygen, peripheral venous access, spontaneous cerebral hemorrhage

## Abstract

**Background:**

Air embolization is usually an iatrogenic complication that can occur in both veins and arteries. Intravenous air embolization is mainly associated with large central vein catheters and mechanical ventilation. A 59-year-old woman was sent to our hospital with spontaneous cerebral hemorrhage and treated conservatively with a left forearm peripheral venous catheter infusion drug. After 48 hours, the patient’s oxygen saturation decreased to 92 % with snoring breathing. Computer tomography of the head and chest revealed scattered gas in the right subclavian, the right edge of the sternum, the superior vena cava, and the leading edge of the heart shadow.

**Methods:**

She was sent to the intensive care unit for high-flow oxygen inhalation and left-side reclining instantly. As the patient was at an acute stage of cerebral hemorrhage and did not take the Trendelenburg position.

**Results:**

The computed tomography (CT) scan after 24 hours shows that the air embolism subsides.

**Conclusion subsections:**

Air embolism can occur in any clinical scenario, suggesting that medical staff should enhance the ability to identify and deal with air embolism. For similar cases in clinical practice, air embolism can be considered.

## 1. Introduction

Air embolism is a clinical feature of a series of diseases caused by air entering the arterial or venous system. Most of the time, air embolism is iatrogenic.^[1]^ It most commonly seen in the placement and removal of venous catheter devices, surgery, radiological examinations, etc. It also has been described as a complication of penetrating injury, labor, diving, and other conditions. Air emboli that form only through a peripheral intravenous line are uncommon.

We report a case of venous air embolism during conservative treatment of spontaneous cerebral hemorrhage. This case reminds medical staff that peripheral venous air embolism can occur in various clinical scenarios, and attention should be paid to patients with blurred consciousness who use peripheral venous pathway infusion.

### 1.1. Case study

A 59-year-old woman was admitted to our hospital with dizziness and headache for 2 hours. She had high blood pressure and diabetes, had taken her own antihypertensive medication, had long-term insulin injections, an unknown dose, and had cataract and appendix surgery. At the time of admission, her blood pressure was 182/98 mm Hg, heart rate 60 per second, body temperature 36.0°C, breathing 19 per second, oxygen saturation 98%. Physical checkup blurred consciousness, glasgow coma scale14 points, The large isospheric diameter of the bilateral pupil is 2.5 mm, Light reflex sensitivity, right limb muscle force level III, Left limb muscle force level V, and head computed tomography (CT) shows: The hematoma of the basal region on the left side broke into the ventricle. She was diagnosed with cerebral hemorrhage and admitted to neurosurgery for treatment.

**Figure 1.**
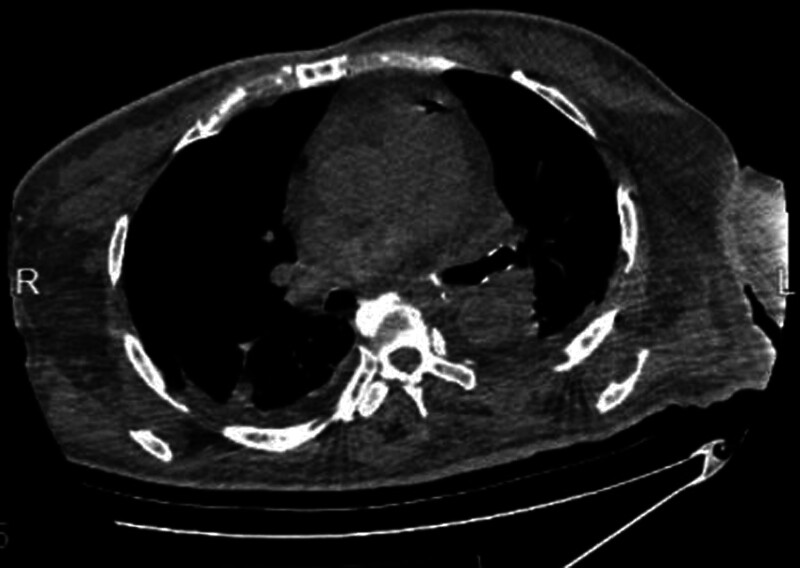
Pulmonary aorta air shadow.

**Figure 2.**
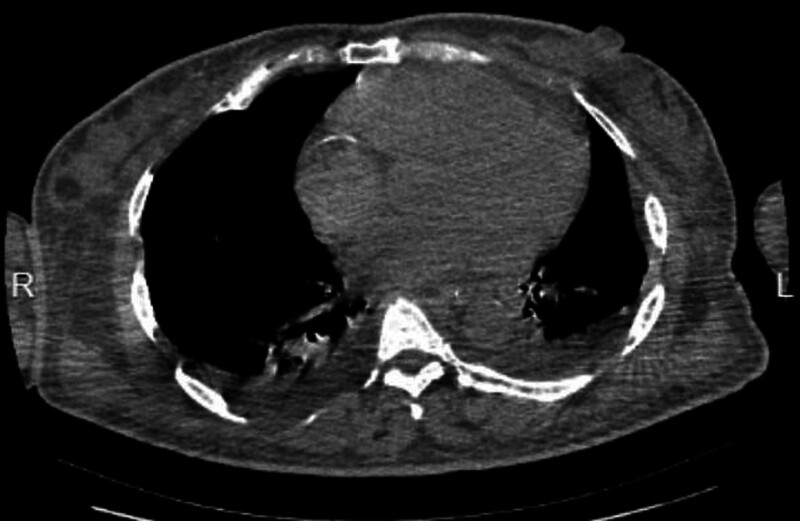
Air embolism subsided.

She was given special care, electrocardiogram, oxygen, nasal feeding and blood glucose monitoring. Her left forearm retains an intravenous retention needle (24G) while using a trace pump for intravenous injection. After 48 hours of admission, her state of consciousness decreased, oxygen saturation decreased to 92%, snoring breathing, and tongue falling appeared. The medical staff did not improve his symptoms after turning over and patting his back. Blood gas analysis showed that pondus hydrogenii 7.41, oxygen partial pressure 68.4 mm Hg, carbon dioxide partial pressure 46.1 mm Hg. Computer tomography of the head and chest revealed scattered gas in the right subclavian, the right edge of the sternum, the superior vena cava, and the leading edge of the heart shadow (Fig. 1). She was immediately transferred to intensive care unit for advanced life support. She began receiving high-flow oxygen, lying on the left side, as the patient was in an acute stage of cerebral hemorrhage and did not take a head-low high position. After 24 hours, another imaging examination showed that the air embolism subsided (Fig. 2). The patient’s vital signs were stable and brain CT showed that cerebral hemorrhage was absorbed.

A series of measures such as supplemental nutrition, anti-infection, anticoagulation, rehabilitation and exercise were given to treat cerebral hemorrhage. After 1 month, the patient recovered and was discharged. No incident occurred during hospitalization.

## 2. Discussion

Air embolism has been reported in a variety of surgeries and clinical procedures since it was first discovered and reported in the 19th century. Because its clinical symptoms and signs are not typical, Some asymptomatic patients are missed and the true incidence of air embolism is unclear.^[2]^ The occurrence of air embolism requires a certain pressure difference between the blood and the atmosphere and the entry of gas into the vascular system, which migrates to different parts of the body as the blood circulates. Arterial air embolization is common in bronchoscopy, craniotomy and other invasive operations, entering the artery circulation of the air clogged carotid arteries and coronary arteries may immediately cause stroke or heart-related symptoms. Intravenous air embolization is due to the placement and extraction of the intravascular catheter, the gas into the venous system as the circulation of blood through the right heart to the pulmonary circulation, causing the proportion of pulmonary ventilation, leading to hypoxemia. Gases can also cause endothelial damage in blood vessels, which can eventually lead to pulmonary edema. In addition, the air in the venous circulation may enter the arterial circulation through the channels of the right-to-left diversion of the heart, such as the patent foramen ovale and atrial septal defect.^[3,4]^

The clinical manifestation of air embolism depends to a large extent on the amount of gas that enters the circulation, the speed at which the embolization occurs, from asymptomatic to circulatory failure and even death. A small amount of air can be dispersed to the alveolar capillaries that bind to hemoglobin or disperse to the alveoli to be discharged by respiration. Patients can be asymptomatic and symptomatic, or have mild headaches, breathing difficulties, nausea, etc. So it is difficulty to diagnose.^[5]^ As the amount and speed of gas entering increases, Air embolization can cause circulatory failure and multi-organ ischemia and hypoxia. It has been reported that the accumulation of gases into the vascular system at 200 to 300 mL can cause death in adults.^[6]^

Early detection and subsequent timely treatment are essential to reduce morbidity and mortality. Physical examination of the “grinding wheel murmur” of the chest hearing, is an auditory sound caused by gas agitation in the heart cavity. It is the only specific sign of an intravenous gas embolism.^[7]^ In addition to detailed inquiries about the patient’s medical history and physical examination, sensitive examination methods are required. For intubated patients, a decrease in the level of carbon dioxide at the end of exhalation is an early sign of air embolism. However, screening with hyperventilation, low cardiac output and chronic obstructive pulmonary disease is required.^[8]^ Esophageal echocardiography is the most definitive diagnostic method for monitoring air in the heart cavity. Detection of air as low as 0.02 mL/kg.^[9]^ Chest X-rays contribute to rapid diagnosis of venous air embolism.^[10]^ Our patients are blurred in consciousness and the diagnosis is determined by changes in hemodynamics and by adjuvant tests.

The first step in the treatment of venous air embolism is to stop the gas from continuing into the blood vessels by closing the pump or clamping the port. Oxygen inhalation at concentrations close to 100% helps increase blood oxygen saturation in the arteries. Reduce nitrogen content and promote the absorption of air embolism.^[11]^ For patients with severe hemodynamic instability of embolization, Hyperbaric oxygen therapy facilitates compression and elimination of bubbles, thereby reducing blood vessel blockage and improving cerebral perfusion.^[12,13]^ The patient’s posture is also important, The left and Trendelenburg confine the bubble to the right ventricular tip, reduces the production of foam blood and relieves air locking between the pulmonary artery and the right ventricle.^[14,15]^ However, the clinical situation is complex and the position should be taken according to the actual situation of the patient. Our patients are in an acute stage of cerebral hemorrhage and do not take the Trendelenburg position.

## 3. Conclusion

Air embolism caused by peripheral superficial vein catheterization is very rare. The patient only established a left forearm venous pathway upon admission and had no history of other invasive operations, suggesting that the venous pathway was the most likely source of air embolism. The reason may be: the presence of air in the intravenous infusion line; the infusion pump provides power to the air into the blood vessel; The pipe is not tightly connected, causing air to enter. Air embolism after peripheral vein pathways may occur in various clinical scenarios, suggesting that caregivers should strengthen the management of infusion lines and pay more attention to intravenous air embolization. For patients who are being treated with peripheral venous pathways, but with blurred minds, the observation should be strengthened. The trace pump extension tube and infusion line connection should be preceded by exhaustion of air and a tight connection. Medical staff should have the ability to quickly identify venous air embolism to avoid irreversible damage due to missed diagnosis. In the future, venous air embolism should be considered in patients who are found to be similar to this case.

## Author contributions

**Conceptualization:** Xingming Zhong, Liying Dong.

**Formal analysis:** Xiaoxiao Zhou.

**Methodology:** Xingming Zhong.

**Writing – original draft:** Xiaoxiao Zhou.

**Writing – review & editing:** Liying Dong.

## Supplementary Material


